# Supplementary material for: A Hox-TALE regulatory circuit for neural crest patterning is conserved across vertebrates
Source: Nat Commun. 2019 Mar 13;10:1189. doi: 10.1038/s41467-019-09197-8 (PMC6416258; doi:10.1038/s41467-019-09197-8)
Supplement: Supplementary file 1 — Supplementary Information [file 41467_2019_9197_MOESM1_ESM.pdf]

A Hox-TALE regulatory circuit for neural crest patterning is conserved across vertebrates

Parker *et al*.

Supplementary Information

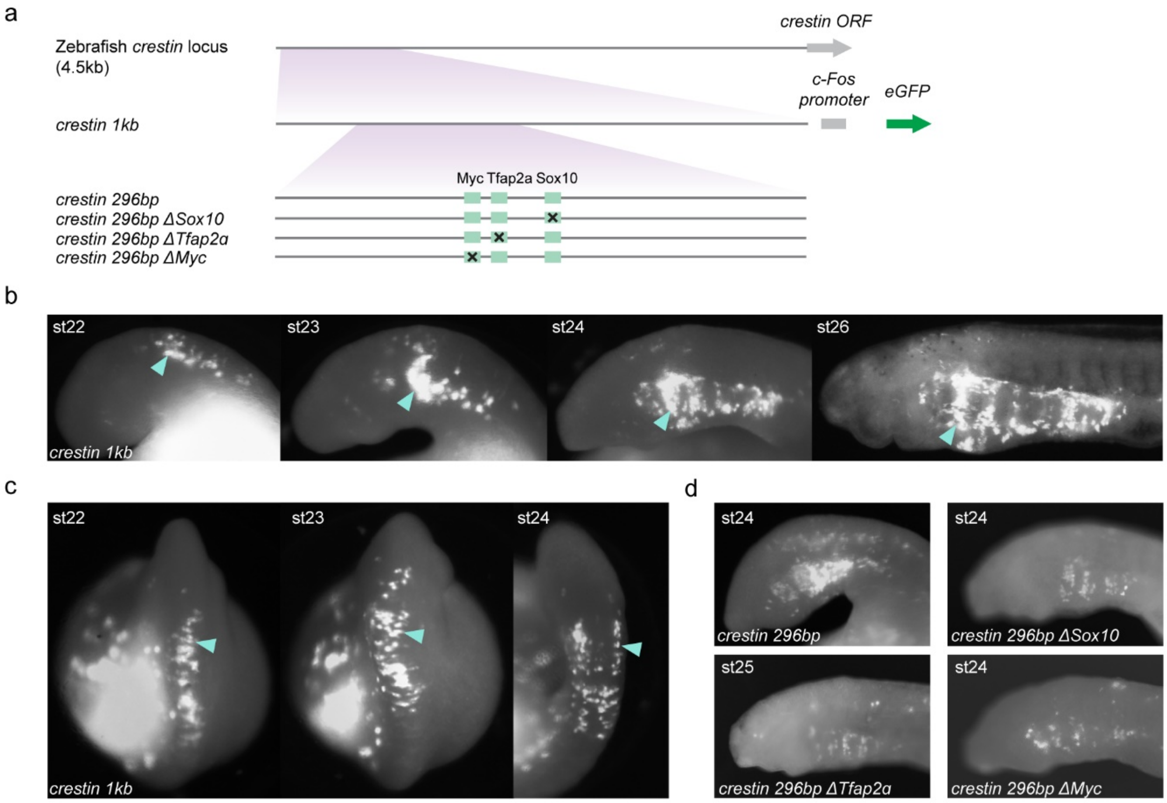

Supplementary Figure 1: GFP reporter expression in NC mediated by variants of the zebrafish *crestin* promoter/enhancer in transient transgenic lamprey embryos.

a, Versions of the zebrafish *crestin* promoter/enhancer tested for activity in zebrafish and lamprey embryos in this study. In zebrafish, the NC-specific activity of the *crestin* element depends upon consensus transcription factor binding sites for multiple transcription factors that are known to be part of a core NC-GRN, including Sox10, Tfap2α and cMyc. Variants of the core minimal promoter/enhancer (*crestin 296bp*) were generated with mutations in these sites (*ΔSox10*, *ΔTfap2α*, *ΔMyc*). Regulatory elements were cloned upstream of the mouse *c-Fos* promoter. **b-c**, Lateral (b) and dorsal (c) views of two different transient transgenic lamprey embryos exhibiting GFP expression in NC (arrowheads) under the control of the *crestin 1kb* promoter/enhancer. Expression is first seen in the dorsal neural tube in NC cells as they start to delaminate, which then migrate to populate the pharyngeal arches at later stages. Even though the *crestin* element is specific to zebrafish and is not present in other gnathostomes, its *cis*-regulatory activity is conserved between lamprey and zebrafish. These data suggest that upstream regulatory factors that mediate activity of the *crestin* element in zebrafish may also be present in lamprey NC. **d**, Lateral views of st24 lamprey embryos injected with variants of the *crestin* promoter/enhancer. A minimal *crestin* promoter/enhancer is active in the lamprey NC; we found that the activity of this element in lamprey is also sensitive to perturbation of the same binding sites critical for activity in zebrafish. These results, coupled with the endogenous expression of *SoxE1-3*, *Tfap2,* and *n-Myc* in lamprey, suggest that the *crestin* element is interpreted in lamprey by components of an ancestral NC GRN that includes Sox, Tfap2α and Myc factors. GFP-expressing embryos shown are representative of the expression potential of the reporter construct in each case, as inferred from screening many (typically more than 100) injected embryos. Supplementary Tables 1-2 provide the injection statistics for *crestin* constructs in zebrafish and lamprey embryos.

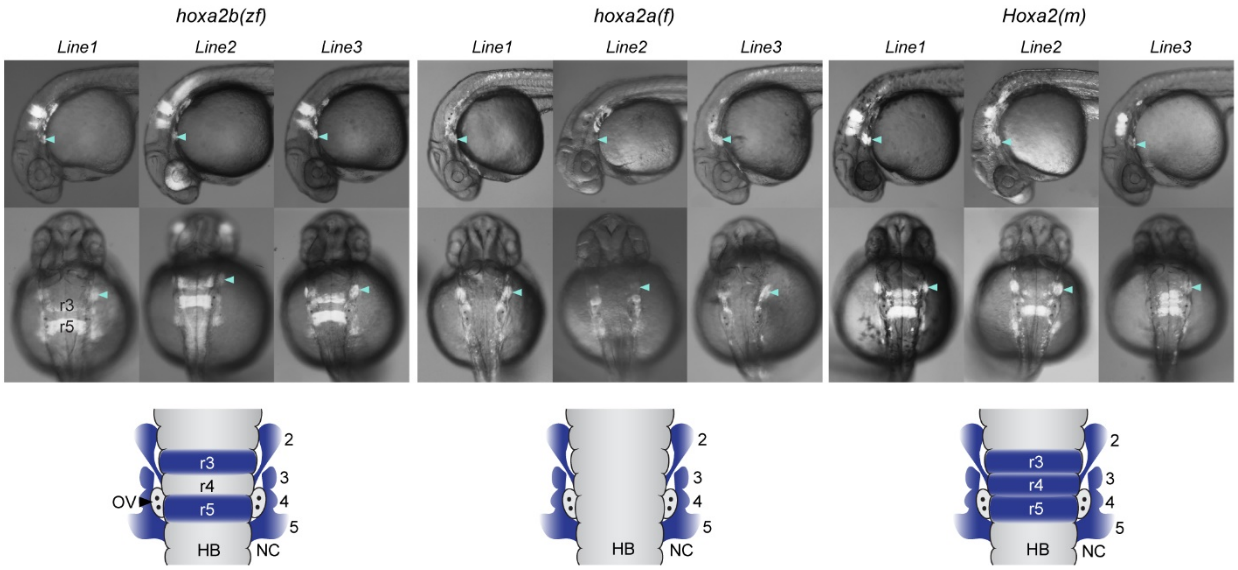

Supplementary Figure 2: GFP reporter expression driven by gnathostome *Hoxa2* NC enhancers in transgenic zebrafish reporter lines.

Three independent lines are shown for each of the three gnathostome *Hoxa2* enhancers. Letters in parenthesis indicate species of origin of the enhancer: zf, zebrafish; f, fugu; m, mouse. Lateral (top) and dorsal (middle) views are shown. Arrowheads denote GFP expression in PA2. Schematics at the bottom depict dorsal views of the rhombomeres (r) and pharyngeal arches (numbered), illustrating the consistent domains of activity observed between separate transgenic lines for each enhancer (blue shading). Abbreviations: HB, hindbrain; NC, neural crest; OV, otic vesicle.

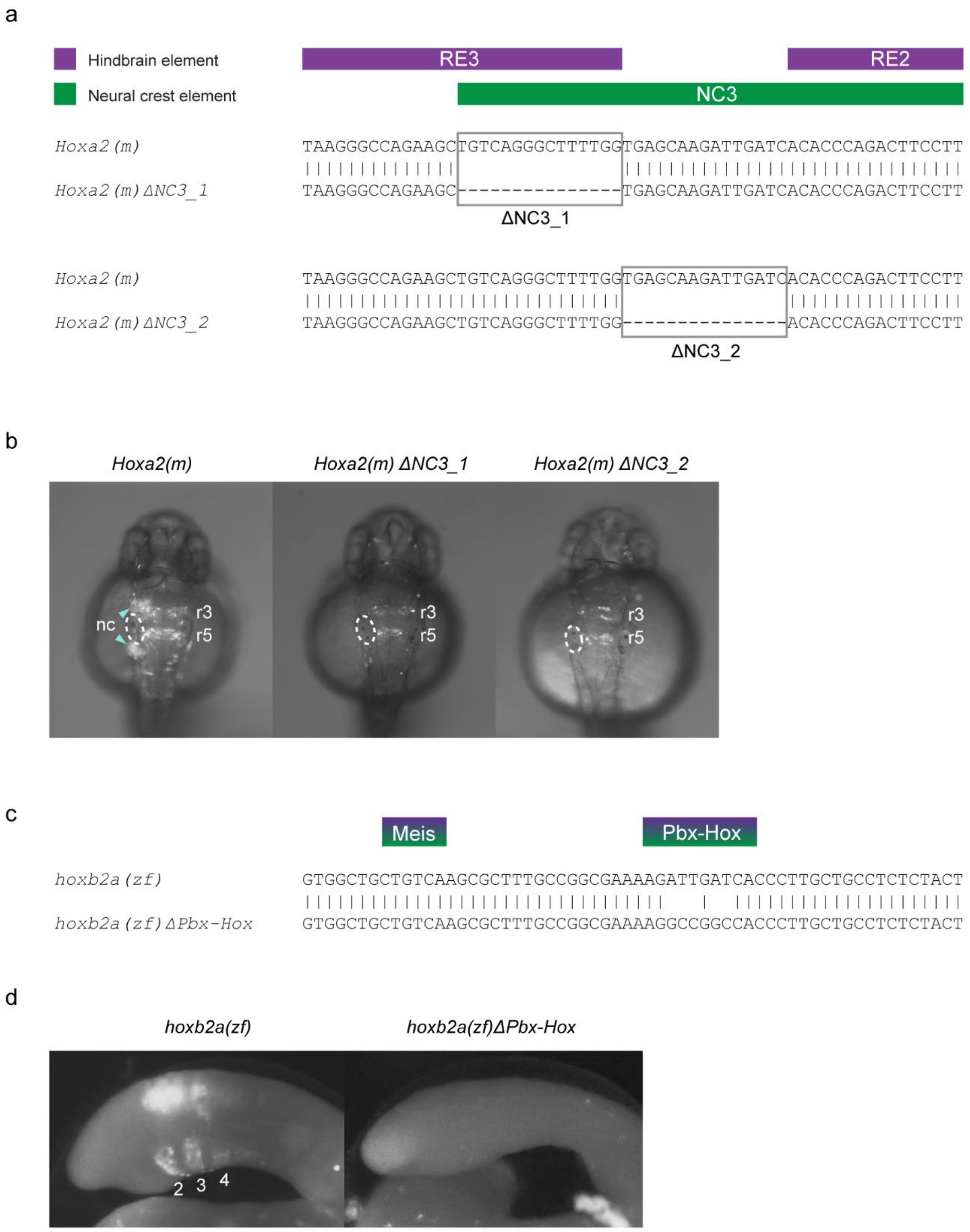

Supplementary Figure 3: Mutation of sites within gnathostome *Hox2* enhancers and their influence on tissue-specific activity in transient transgenic zebrafish and lamprey embryos.

a, Alignments of the *NC3* region of the wild-type mouse (m) *Hoxa2* NC enhancer with two variants, *ΔNC3_1* and *ΔNC3_2*, showing the 15bp portions deleted in each variant. The positions of characterised hindbrain

(purple) and NC (green) *cis*-elements are shown above the alignments. **b**, Dorsal views of transient transgenic zebrafish embryos with GFP expression mediated by the wild-type *Hoxa2(m)* enhancer and the two *ΔNC3* variants. The left otic vesicle of each embryo is circled, with GFP expression in rhombomeres (r) and neural crest (nc) annotated. Embryos are at approximately 30 hours post-fertilisation. **c**, Alignment of a portion of the wild-type zebrafish (zf) *hoxb2a* NC enhancer with a variant in which the Pbx-Hox site has been mutated (*ΔPbx-Hox*). **d**, st24 transient transgenic lamprey embryos injected with the wild-type *hoxb2a(zf)* (lateral view) and mutated *hoxb2a(zf)ΔPbx-Hox* (dorsal view) enhancers. *hoxb2a(zf)* mediates expression in the hindbrain and neural crest, posterior to PA1 (pharyngeal arches are numbered), while this activity is lost in *hoxb2a(zf)ΔPbx-Hox.* GFP-expressing embryos shown in **b** and **d** are representative of the expression potential of the reporter construct in each case, as inferred from screening many (typically more than 100) injected embryos. The injection statistics for *Hoxa2(m)* and mutated variants in transient transgenic zebrafish embryos are provided in Supplementary Table 3, while those for the *hoxb2a(zf)* constructs in lamprey embryos are given in Supplementary Table 2.

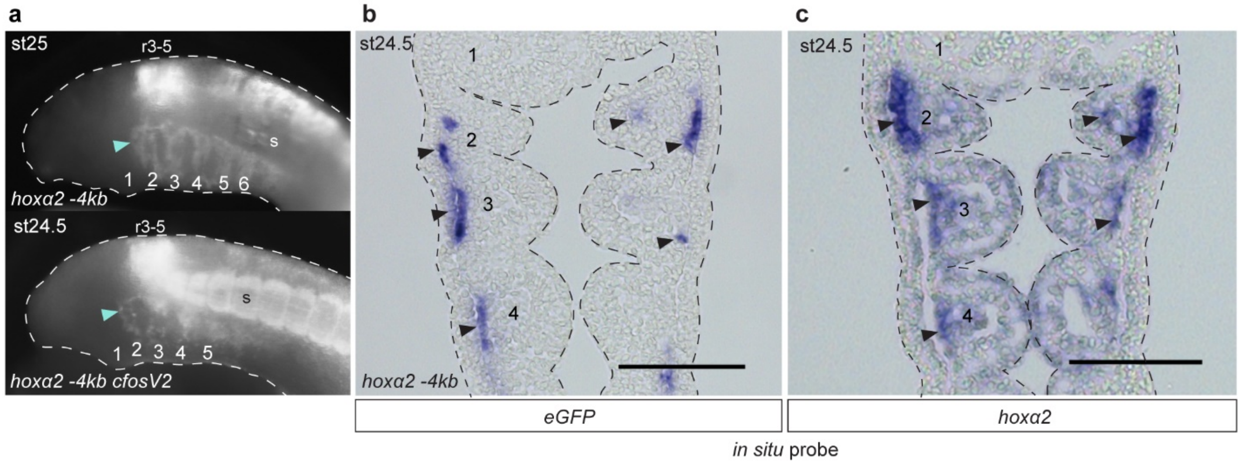

Supplementary Figure 4: GFP reporter expression driven by lamprey *hoxα2* upstream regions in transient transgenic lamprey embryos.

a, Lateral views of st24-25 transient transgenic lamprey embryos showing GFP expression in rhombomeres (r), somites (s) and NC of the pharyngeal arches (numbered), driven by the *hoxα2 -4kb* enhancer with or without the mouse *c-Fos* minimal promoter. In cloning the *c-Fos* promoter between the lamprey enhancer and the GFP coding sequence, two alternative reporter constructs were generated: *hoxα2 -4kb cFosV1* with the upstream lamprey sequence fully intact, *hoxα2 -4kb cFosV2* with the 5'UTR partially removed. In both cases, the insertion of the *c-Fos* promoter increased levels of reporter expression but did not influence tissue-specific expression domains. GFP-expressing embryos shown are representative of the expression potential of the reporter construct in each case, as inferred from screening many (typically more than 100) injected embryos (see Supplementary Table 2 for expression statistics). **b**, Frontal section through a transient transgenic lamprey embryo at st24.5, revealing GFP transcripts in the NC-derived mesenchyme (arrows) of the pharyngeal arches (numbered), driven by *hoxα2 -4kb*. **c**, Frontal section through a st24.5 lamprey embryo showing endogenous *hoxα2* expression. Arrows indicate elevated expression in the NC-derived mesenchyme. The pharyngeal arches are numbered. Scale bars: 100µm. Abbreviations: r, rhombomere; s, somites.

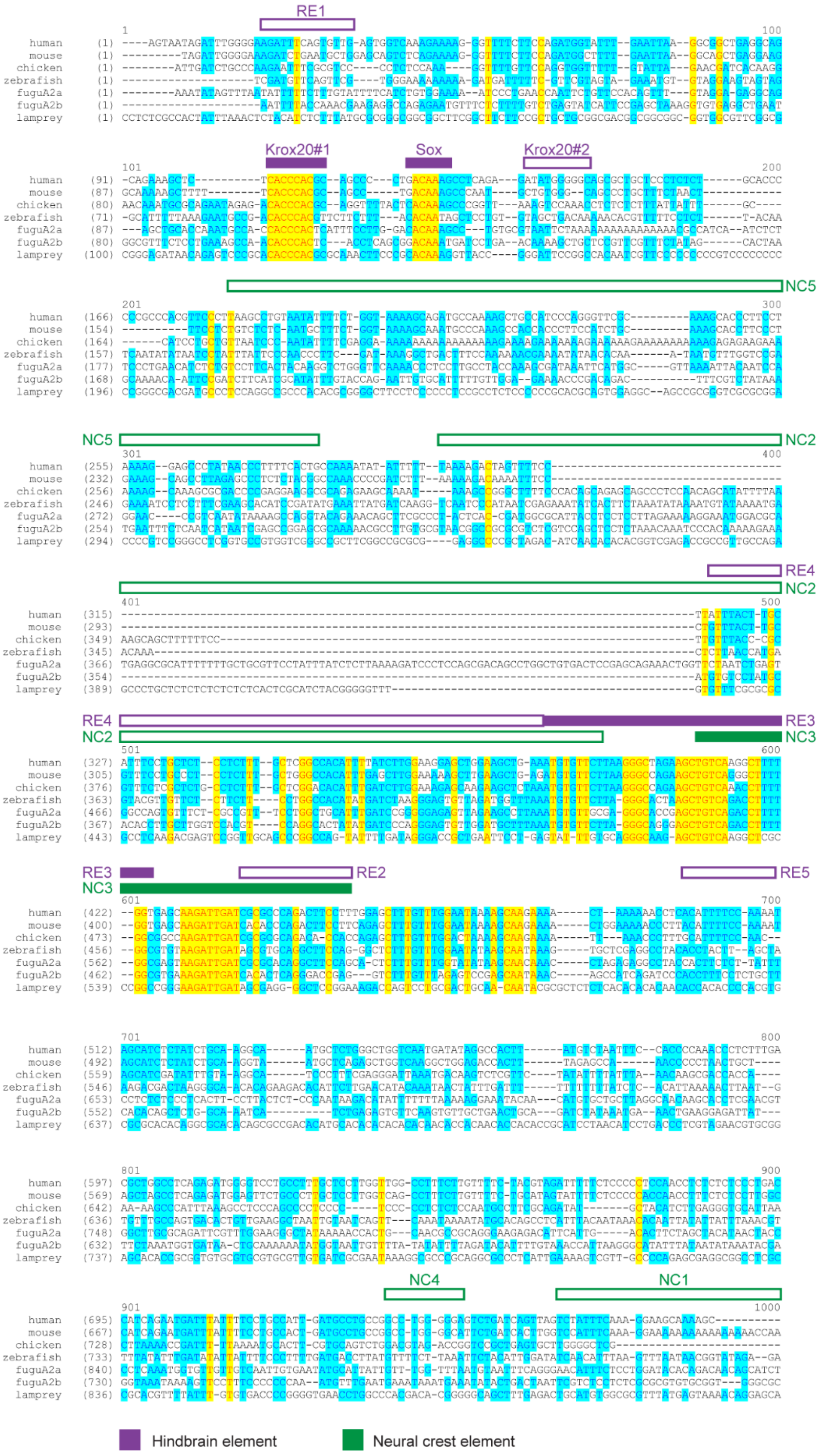
RE1
Krox20#1
Sox
Krox20#2
NC5
NC2
RE4
RE3
NC3
RE2
RE5
NC4
NC1
human
mouse
chicken
zebrafish
fuguA2a
fuguA2b
lamprey
Hindbrain element
Neural crest element

Supplementary Figure 5: Multiple sequence alignment of gnathostome *Hoxa2* NC enhancers and the lamprey *hoxα2* enhancer.

Positions with sequence conservation across all species in the alignment are shaded in yellow, while blue shading denotes conservation across more than half of the species at that position. Characterised *cis*-elements from mouse that contribute to hindbrain (purple) or NC (green) activities are indicated above the alignment. Sequence conservation of these elements in lamprey is denoted by a shaded box (such as *Krox20#1*), while sequence divergence is indicated by an empty box (such as *RE1*). Sequence conservation between gnathostomes and lamprey is only seen for short sequences corresponding to the *Krox20#1*, *Sox*, and *RE3/NC3* elements. A portion of this alignment is shown in **Fig.4f**.

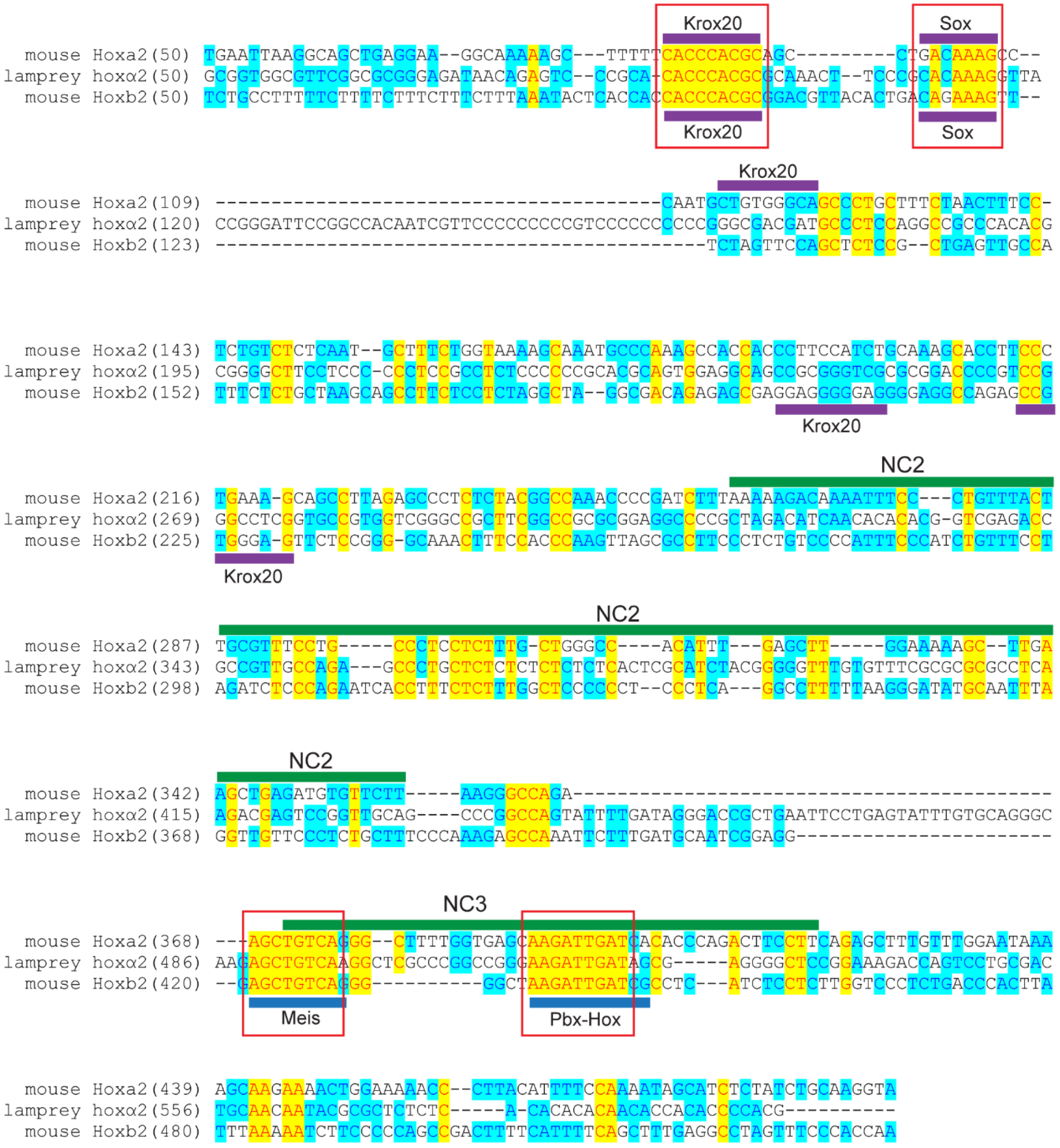

Supplementary Figure 6: Multiple sequence alignment of mouse *Hoxa2* and *Hoxb2* NC enhancers with the lamprey *hoxα2* enhancer.

Positions with sequence conservation across all enhancers in the alignment are shaded in yellow, while blue shading denotes conservation across more than half of the enhancers at that position. Characterised *cis*-elements from the mouse *Hoxa2* enhancer are shown above the alignment and from *Hoxb2* below. The sequences conserved between all three enhancers are outlined by red boxes and correspond to *Krox20*, *Sox* and *NC3/Meis/Pbx-Hox cis*-elements. A portion of this alignment was shown in **Fig.5a**.

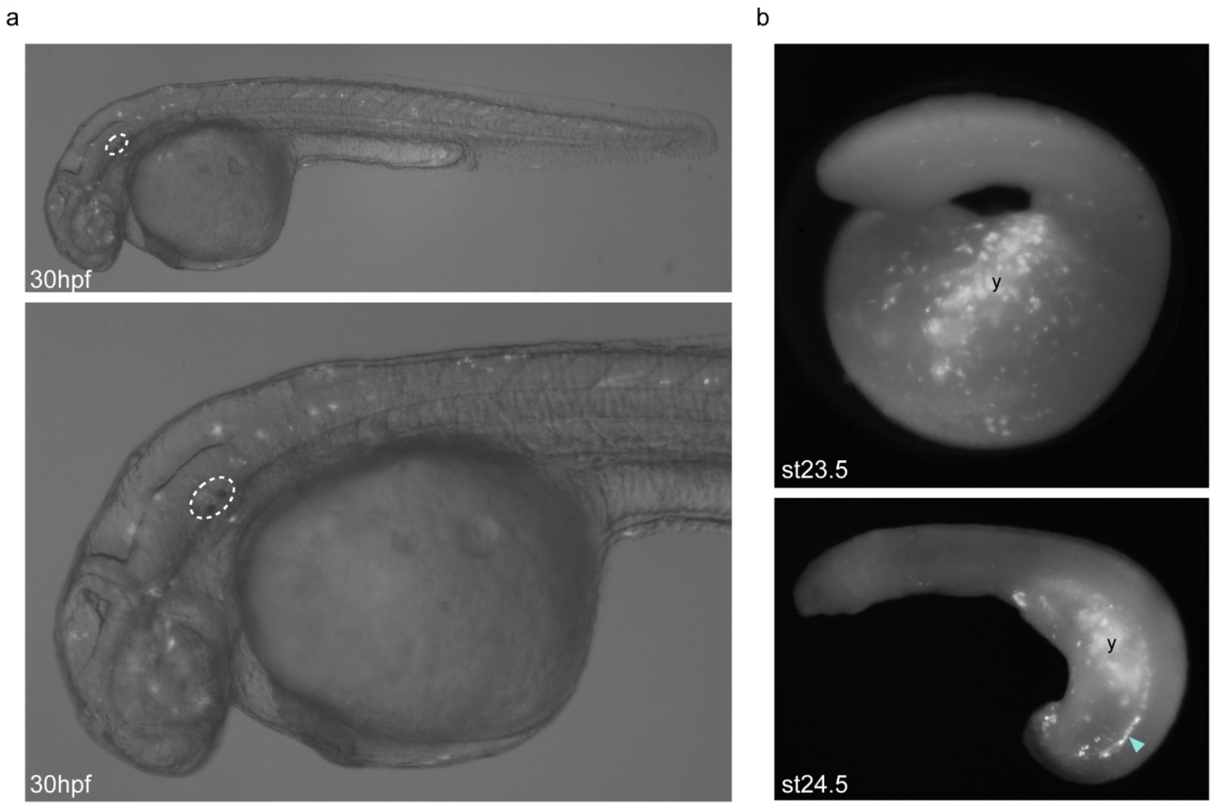

Supplementary Figure 7: Background GFP expression driven by the empty HLC vector in zebrafish and lamprey embryos.

a, Lateral views of 30hpf/prim-16 stage transient transgenic zebrafish embryos injected with the empty HLC vector using *Tol2*-mediated transgenesis, showing low-intensity mosaic GFP expression in multiple tissue types including neurons and muscle cells. The otic vesicle is circled. **b**, Lateral views of st23.5 and st24.5 transient transgenic lamprey embryos injected with the empty HLC vector using I-SceI-mediated transgenesis, showing mosaic GFP expression in the yolk (y) and ectoderm, as well as cells lying dorsal to the yolk (arrowhead). GFP-expressing embryos shown are representative of the expression potential of the reporter construct in each case, as inferred from screening many (typically more than 100) injected embryos.

Supplementary Table 1: Reporter assay statistics for the zebrafish *crestin* minimal promoter/enhancer and mutated variants in zebrafish and lamprey embryos.

	Zebrafish embryos				Lamprey embryos			
Element[1]	# Embryos	# GFP +ve[2]	# NC expression	%NC expression	# Embryos	# GFP +ve	# NC expression	%NC expression
crestin296bp	159	70	66	94.3	138	99	57	57.6
crestin296bpΔSox10[3]	111	78	10	12.8	100	94	17	18.1
crestin296bpΔTfap2α	52	47	8	17.0	301	279	81	29.0
crestin296bpΔMyc	138	86	7	8.1	549	460	72	15.7

[1] For each injected construct the total number of screened embryos and the number exhibiting GFP expression in neural crest cells (NC) are given. In each case, the numbers derive from individual rounds of injection.

[2] The total number of GFP-expressing (GFP +ve) embryos is given, which reflects injection efficiency since transient transgenic embryos often express GFP in additional non-specific domains. The proportion of these GFP +ve embryos with NC expression is calculated (%NC expression).

[3] Variants of the minimal promoter/enhancer have mutations in consensus transcription factor binding-sites for either Sox10, Tfap2α, or c-Myc (*ΔSox10*, *ΔTfap2α*, *ΔMyc*).

Supplementary Table 2: Lamprey reporter assay statistics.

Element[1]	# embryos	# pharyngeal expression	% pharyngeal expression	# hindbrain expression	% hindbrain expression	# somite expression	% somite expression
crestin_1kb (zf)	165	75	45.5	0	0.0	0	0.0
Hoxa2(m)	170	95	55.9	73	42.9	41	24.1
hoxa2b(zf)	132	97	73.5	29	22.0	7	5.3
hoxa2a(f)	137	125	91.2	83	60.6	110	80.3
Hoxa2(m)ΔNC3_1	153	3	2.0	19	12.4	0	0.0
Hoxa2(m)ΔNC3_2	220	0	0.0	49	22.3	0	0.0
hoxb2a(zf)	192	N/A[2]	N/A	113	58.9	N/A	N/A
hoxb2a(zf)ΔPbx-Hox	383	0	0.0	0	0.0	0	0.0
hoxα2 -4kb	288	57	19.8	16	5.6	78	27.1
hoxα2 -4kb cfosV1	254	98	38.6	61	24.0	122	48.0
hoxα2 -4kb cfosV2	220	145	65.9	80	36.4	161	73.2
hoxα2 -4kb Δkrox20	293	164	56.0	79	27.0	209	71.3
hoxα2 -4kb ΔNC3	249	14	5.6	0	0.0	198	79.5
hoxα2 -4kb elementA	181	130	71.8	89	49.2	107	59.1

[1] For each injected construct, the tissue-specific GFP expression domains are given, along with the number and proportion of screened embryos exhibiting GFP expression in those domains. In each case, the numbers derive from individual rounds of injection. Letters in parentheses after the element names indicate the species of origin of the element: f, fugu; m, mouse; zf, zebrafish.

[2] N/A numbers on efficiency not available.

Supplementary Table 3: Zebrafish transient reporter assay statistics for *Hoxa2(m)* and mutated variants.

Element[1]	# embryos	# GFP +ve[2]	# pharyngeal expression	% pharyngeal expression	# hindbrain expression	% hindbrain expression
Hoxa2(m)	72	63	42	66.7	42	66.7
Hoxa2(m)ΔNC3_1	111	101	3	3.0	61	60.4
Hoxa2(m)ΔNC3_2	119	70	0	0	26	37.1

[1] For each injected construct the total number of screened embryos and the number exhibiting GFP expression in specific tissue domains are given. In each case, the numbers derive from individual rounds of injection.

[2] The total number of GFP-expressing (GFP +ve) embryos is given, which reflects injection efficiency since transient transgenic embryos often express GFP in additional non-specific domains. The proportions of these GFP +ve embryos with hindbrain and pharyngeal expression are calculated.
