# Supplementary material for: A Hox-TALE regulatory circuit for neural crest patterning is conserved across vertebrates
Source: Nat Commun. 2019 Mar 13;10:1189. doi: 10.1038/s41467-019-09197-8 (PMC6416258; doi:10.1038/s41467-019-09197-8)
Supplement: Supplementary file 3 — Reporting Summary [file 41467_2019_9197_MOESM3_ESM.pdf]

nature research

Corresponding author(s): *Prof Robb Krumlauf*

Reporting Summary

Nature Research wishes to improve the reproducibility of the work that we publish. This form provides structure for consistency and transparency in reporting. For further information on Nature Research policies, see Authors & Referees and the Editorial Policy Checklist.

Statistical parameters

When statistical analyses are reported, confirm that the following items are present in the relevant location (e.g. figure legend, table legend, main text, or Methods section).

n/a	Confirmed	
☐	☒	The exact sample size (*n*) for each experimental group/condition, given as a discrete number and unit of measurement
☒	☐	An indication of whether measurements were taken from distinct samples or whether the same sample was measured repeatedly
☒	☐	The statistical test(s) used AND whether they are one- or two-sided<br>*Only common tests should be described solely by name; describe more complex techniques in the Methods section.*
☒	☐	A description of all covariates tested
☒	☐	A description of any assumptions or corrections, such as tests of normality and adjustment for multiple comparisons
☒	☐	A full description of the statistics including central tendency (e.g. means) or other basic estimates (e.g. regression coefficient) AND variation (e.g. standard deviation) or associated estimates of uncertainty (e.g. confidence intervals)
☒	☐	For null hypothesis testing, the test statistic (e.g. *F*, *t*, *r*) with confidence intervals, effect sizes, degrees of freedom and *P* value noted<br>*Give P values as exact values whenever suitable.*
☒	☐	For Bayesian analysis, information on the choice of priors and Markov chain Monte Carlo settings
☒	☐	For hierarchical and complex designs, identification of the appropriate level for tests and full reporting of outcomes
☒	☐	Estimates of effect sizes (e.g. Cohen's *d*, Pearson's *r*), indicating how they were calculated
☒	☐	Clearly defined error bars<br>*State explicitly what error bars represent (e.g. SD, SE, CI)*

Our web collection on statistics for biologists may be useful.

Software and code

Policy information about availability of computer code

Data collection	Sequencing of ChIP-seq libraries was performed on the Illumina HiSeq 2500, 51bp single-end. Raw reads were aligned to the UCSC mm10 mouse genome with bowtie2 2.2.0 12. Primary reads from each bam were normalized to reads-per-million and bigWig tracks visualized at the UCSC genome browser (https://genome.ucsc.edu/)<br>ATAC-seq data was sequenced on Illumina HiSEq. 2500 instrument. Following sequencing, Illumina Real Time Analysis v1.18.64 and CASAVA v1.8.2 were run to demultiplex reads and generate FASTQ files.
Data analysis	*Provide a description of all commercial, open source and custom code used to analyse the data in this study, specifying the version used OR state that no software was used.*

For manuscripts utilizing custom algorithms or software that are central to the research but not yet described in published literature, software must be made available to editors/reviewers upon request. We strongly encourage code deposition in a community repository (e.g. GitHub). See the Nature Research guidelines for submitting code & software for further information.

Data

Policy information about availability of data

All manuscripts must include a data availability statement. This statement should provide the following information, where applicable:

- Accession codes, unique identifiers, or web links for publicly available datasets
- A list of figures that have associated raw data
- A description of any restrictions on data availability

All raw sequencing data from this study have been submitted to the NCBI BioProject database (https://www.ncbi.nlm.nih.gov/bioproject) under accession number PRJNA341679 and Sequence Read Archive under accession no. SRP079975.
All original source data are deposited in the Stowers Institute Original Data Repository and available online at http://odr.stowers.org/websimr/.

Field-specific reporting

Please select the best fit for your research. If you are not sure, read the appropriate sections before making your selection.

☒ Life sciences ☐ Behavioural & social sciences ☐ Ecological, evolutionary & environmental sciences

For a reference copy of the document with all sections, see nature.com/authors/policies/ReportingSummary-flat.pdf

Life sciences study design

All studies must disclose on these points even when the disclosure is negative.

Sample size	In transgenic regulatory analyses in zebrafish and lamprey embryos, in general a minimum of 100 embryos were injected for each construct. This permits monitoring efficiency and variability for each construct due to mosaicism and position effects of integration.
Data exclusions	No data was excluded from the analyses
Replication	To monitor reproducibility in regulatory analyses a minimum of 100 embryos were injected and results for all embryos scored. In several cases transient expression results in zebrafish were validated by generating three independent stable lines of fish with the same constructs. This enabled testing and reproducibility of reporter expression at any desired developmental stage.
Randomization	Experimental groups were assigned based on DNA constructs used for injection.
Blinding	N/A

Reporting for specific materials, systems and methods

Materials & experimental systems

n/a	Involved in the study	
☒	☐	Unique biological materials
☐	☒	Antibodies
☐	☒	Eukaryotic cell lines
☒	☐	Palaeontology
☐	☒	Animals and other organisms
☒	☐	Human research participants

Methods

n/a	Involved in the study	
☐	☒	ChIP-seq
☒	☐	Flow cytometry
☒	☐	MRI-based neuroimaging

Antibodies

Antibodies used	KH2 ES cells with epitope-tagged Hoxb1 (3XFLAG-MYC) were used for Hoxb1 ChIP using anti-flag antibody (F1804-Sigma). ChIP-seq and ATAC-seq data for TALE proteins, EP300 & ATAC were obtained from previously deposited (NCBI-SRP079975 & NCBI PRJNA341679) and published data sets (DeKumar et al (2017) PNAS 114: 5838-5845 and De Kumar et al (2017) Genome Research, 27:1501-1512) that were generated using unmodified KH2 cell lines and antibodies for Pbx (SC-888; Santacruz), Meis (SC-25412; Santacruz), Prep1 (ab55603; Abcam), Prep2 (sc-292315X; Santacruz) and EP300 (Sc-585X; Santacruz).
Validation	The ChIP-seq and ATAC-seq data sets analyzed in this study were obtained from previously deposited data sets and published experiments (NCBI-SRP079975 & NCBI PRJNA341679; DeKumar et al (2017) PNAS 114: 5838-5845 and De Kumar et al (2017)

Genome Research, 27:1501-1512). These experiments used antibodies from commercial sources, validated according to manufacturers specifications and used in previously published experiments.

Eukaryotic cell lines

Policy information about cell lines

Cell line source(s)	KH2 mouse embryonic stem cells ES cells were obtained from Open Biosystems (Catalog #MES 4304). Lot #KH2060
Authentication	KH2 ES cells were authenticated by PCR and sequencing the specific targeted integration site. The cells were karyotyped and used in blastocyst injections to show that they could generate lines of mice.
Mycoplasma contamination	All lines were tested and validated to be micoplasma free.
Commonly misidentified lines (See ICLAC register)	*Name any commonly misidentified cell lines used in the study and provide a rationale for their use.*

Animals and other organisms

Policy information about studies involving animals; ARRIVE guidelines recommended for reporting animal research

Laboratory animals	For laboratory zebrafish, the wild-type Slusarski AB zebrafish line was used for embryo micro-injection experiments using Tol2-mediated transgenesis. Eggs were harvested from matings of males and females and injections were done in 1-2 cell embryos. The following pre-existing zebrafish transgenic reporter lines were used for this study: Tg(dr.hoxa2b:eGFP: Parker, H. J., et al Nature 514, 490-493 (2014)) and Tg(fr.Hoxa2a:eGFP: McEllin, J. A. et al Dev Biol 409, 530-542 (2016)). The line Tg(mm.Hoxa2b:eGFP) was generated in this study from a founder that had been micro-injected with Hoxa2(m)-HLC.<br><br>This study was conducted in accordance with the Guide for the Care and Use of Laboratory Animals of the National Institutes of Health and protocols were approved by the Institutional Animal Care and Use Committees of the Stowers Institute (zebrafish, RK Protocol #2015-0149 and Protocol #2018-0184).
Wild animals	Gravid male and female sea lamprey (Petromyzon marinus) were caught in the wild and provided by the Great Lakes Fisheries Commission (GLFC), in coordination with the USGS Hammond Bay Biological Station, Millersburg, MI, USA. They were sent overnight, in accordance with CA state import permits, in chilled, oxygenated water to the lamprey facility at the California Institute of Technology. They were maintained under the parameters set in accordance with the Guide for the Care and Use of Laboratory Animals of the National Institutes of Health, with protocols approved by the Institutional Animal Care and Use Committees of the California Institute of Technology (lamprey, Protocol #1436-17). After spawning, the captive adult lamprey died of natural causes, as per their semelparous reproductive strategy.
Field-collected samples	Embryos were harvested by in-vitro fertilisation at the California Institute of Technology lamprey facility, using captive gravid lamprey (Petromyzon marinus) obtained from the wild and provided by the Great Lakes Fisheries Commission (GLFC), in coordination with the USGS Hammond Bay Biological Station, Millersburg, MI, USA. Captive gravid lamprey were maintained at an appropriate temperature (10-18 degrees C) and on a 15:9 (Day:Night) photoperiod.

ChIP-seq

Data deposition

☒ Confirm that both raw and final processed data have been deposited in a public database such as GEO.

☒ Confirm that you have deposited or provided access to graph files (e.g. BED files) for the called peaks.

Data access links<br>*May remain private before publication.*	All raw sequencing data from this study have been submitted to the NCBI BioProject database (https://www.ncbi.nlm.nih.gov/bioproject) under accession number PRJNA341679 and Sequence Read Archive under accession no. SRP079975 and and PRJNA503882.
Files in database submission	24-hour RA-induction p300 input, replicate 2 SRX2105326<br>24-hour RA-induction p300 input, replicate 1 SRX2105325<br>24-hour RA-induction p300 IP, replicate 2 SRX2105323<br>24-hour RA-induction p300 IP, replicate 1 SRX2105312<br>24-hour RA-induction Prep1-Prep2-TGIF input SRX2105295<br>24-hour RA-induction TGIF IP, replicate 2 SRX2105294<br>24-hour RA-induction TGIF IP, replicate 1 SRX2105293<br>24-hour RA-induction Prep2 IP, replicate 2 SRX2105292<br>24-hour RA-induction Prep2 IP, replicate 1 SRX2105291<br>24-hour RA-induction Prep1 IP, replicate 2 SRX2105289<br>24-hour RA-induction Prep1 IP, replicate 1 SRX2105288<br>24-hour RA-induction Pbx-Meis input, replicate 2 SRX2105287<br>24-hour RA-induction Pbx-Meis input, replicate 1 SRX2105286<br>24-hour RA-induction Meis IP, replicate 2 SRX2105285<br>24-hour RA-induction Meis IP, replicate 1 SRX2105284<br>24-hour RA-induction Pbx IP, replicate 2 SRX2105283

	24-hour RA-induction Pbx IP, replicate 1 SRX2105282
Genome browser session (e.g. UCSC)	https://genome.ucsc.edu/cgi-bin/hgTracks?db=mm10&lastVirtModeType=default&lastVirtModeExtraState=&virtModeType=default&virtMode=0&nonVirtPosition=&position=chr6%3A52151848%2D52181847&hgsid=684046641_NgftwrjTZ941wYbvOsNxSknwDlwh

Methodology

Replicates	All ChIP Seq experiments were done in Biological Duplicate. Each each biological replicates had three technical replicates which were pooled before library preparation and sequencing.
Sequencing depth	All Sequencing were done as 51bp single end Sequence. Sequencing were done to achieve 30 million reads per biological replicates .
Antibodies	KH2 ES cells with epitope-tagged Hoxb1 (3XFLAG-MYC) were used for Hoxb1 ChIP using anti-flag antibody (F1804-Sigma). Unmodified KH2 cell lines were used for ChIP experiments for Pbx (SC-888; Santacruz), Meis (SC-25412; Santacruz), Prep1 (ab55603; Abcam), Prep2 (sc-292315X; Santacruz) and EP300 (Sc-585X; Santacruz). KH2 ES cells 10 with epitope-tagged Hoxb1 (3XFLAG-MYC) were used for Hoxb1 ChIP using anti-flag antibody (F1804-Sigma). Unmodified KH2 cell lines were used for ChIP experiments for Pbx (SC-888; Santacruz), Meis (SC-25412; Santacruz), Prep1 (ab55603; Abcam), Prep2 (sc-292315X; Santacruz) and EP300 (Sc-585X; Santacruz).
Peak calling parameters	MACS2 2.1.0.20140616, parameters "-p 0.25 –m 5 50". Top 100,000 peaks by p-value, for each replicate, were compared with IDR 1.7.0 (https://sites.google.com/site/anshulkundaje/projects/idr) and valid pairs with IDR p-value ≤ 0.01 were taken as the sample peak list.
Data quality	We used IDR as way to check data quality and consistency between Experiments
Software	N/A
